# Association of the overlap of cognitive impairment and depression with 6-month mortality in hospitalized older adults: results from the Re.Po.SI register

**DOI:** 10.1186/s12877-025-05818-8

**Published:** 2025-03-18

**Authors:** Theresa Westgård, Gianluca Bianco, Alessandro Nobili, Mauro Tettamanti, Alessandra Marengoni, Alberto Zucchelli

**Affiliations:** 1 Centre for Ageing and Health (Agecap), Sahlgrenska Academy, the University of Gothenburg, Gothenburg, Sweden; 2 Department of Occupational Therapy and Physiotherapy, Sahlgrenska University Hospital, Region Västra Götaland, Gothenburg, Sweden; 3 SWETALY - Swedish-Italian University Collaboration with a Focus on Ageing Research, Gothenburg, Sweden; 4 Department of Clinical and Experimental Sciences, University of Brescia, Brescia, Italy; 5 Institute of Pharmacological Research Mario Negri IRCCS, (Istituto di Ricerche Farmacologiche Mario Negri IRCCS, Milano), Milan, Italy; 6 Aging Research Centre, Department Neurobiology, Care Sciences and Society, Karolinska Institute, Stockholm, Sweden

**Keywords:** Older people, Depression, Cognitive impairment, Mortality

## Abstract

**Background:**

When admitted to hospital for unplanned medical needs, the complexity of multiple conditions, including cognitive and mental health, might put older people at greater risk, affecting their survival. This study aimed to investigate the prevalence of cognitive impairment versus cognitive impairment with depression and their association with six-month mortality in older people after an unplanned hospital admission in Italy.

**Methods:**

In Re.Po.SI. a multi-centre study performed in Italy, standardized web-based case report forms were used to collect data on socio-demographic factors, clinical parameters, diagnoses, treatment history and at discharge, clinical events during hospitalization, and outcome data was collected. A comprehensive geriatric assessment was conducted using Cumulative Illness Rating Scale (CIRS), Geriatric Depression Scale (GDS-4), Barthel Index, and Short Blessed Test (SBT). To explore the interrelationship between depression and cognitive impairment, a variable categorized the study population into four mutually exclusive groups. This variable assessed the association between its categories and six-month mortality in a Cox multivariate analysis.

**Results:**

One thousand nine hundred fifty six participants were included, with a median age of 80 years (IQR: 73–85). Those who died within six months were likely to be older (82 vs. 79 years), male (56.2% vs. 47.2%), had moderately reduced ability to perform daily activities (82.0 vs. 93.0), exhibited greater illness severity (CIRS-IS: 1.8 vs. 1.6), had more chronically prescribed medications (6.0 vs. 5.0), and had a worse SBT score (10.0 vs. 7.0). When stratified based on cognitive impairment and depression, one-third had neither condition (33.2%), 21.9% had depression, 20.7% had a cognitive impairment, and 24.3% had both conditions. Six-month mortality was higher among people with cognitive impairment only (33.2%) followed by those with both conditions (28.8%), and depression only (22.7%). The unadjusted semi-parametric survival analysis revealed that the hazard ratio (HR) for people with cognitive impairment only was 2.08, for those with both conditions HR was 1.75, and for people with depression only HR was 1.30.

**Conclusion:**

While depression alone may contribute to mortality risk, cognitive impairment appears to play a more substantial role in increasing the risk of dying within 6 month from an acute hospitalization. Further research is needed to confirm these finding.

**Supplementary Information:**

The online version contains supplementary material available at 10.1186/s12877-025-05818-8.

## Background

The Italian population is aging and living longer. Along with this trend, clinicians will meet more, older people with complex and multiple chronic diseases. Older people admitted to hospital for unplanned medical needs will often need longer hospital stays and are at higher risk of mortality [1]. Depression is commonly found in older physically ill patients in general hospitals, but risk estimates vary widely (5–58%), and has been found to unfavourably affect the outcome of numerous medical conditions, reduces treatment compliance, impairs rehabilitation, and decrease survival [2]. Similarly, cognitive impairment is highly prevalent in acute care environments and commonly indicates a poorer prognosis in hospitalized older people [3], since memory loss, slower thinking skills, decreased concentration and ability to make decisions affect capabilities in everyday life [4]. However, a gap remains in the research and literature if the interplay between the two affects mortality in older people after an unplanned hospital admission.

The aim of this study was to investigate the prevalence of cognitive impairment versus cognitive impairment with depression and their association with mortality after 6 months in older people admitted to hospital for unplanned medical needs.

## Material and methods

### Study design

The Re.Po.SI [5] is a collaborative effort between the Italian Society of Internal Medicine (SIMI) and the Mario Negri Institute of Pharmacological Research (Milan). The Re.Po.SI registry was designed to function as a network of internal medicine and geriatric wards evaluating patients affected by multiple diseases and prescribed with polypharmacy. Participation in the network was on a voluntary basis, but choosing to be a participating centre, has increased the attention given to their homogeneous composition in terms of geographic distribution, size, and unselected admissions from the territory or the emergency department. All the patients admitted to the wards participating in the study were recruited consecutively if they were 65 years old or older. Participation in the study was voluntary and all the patients signed an informed consent. The first wave of the Re.Po.SI register was held between January and December 2008 [6]. A standardized web-based case report form was filled in by the attending physicians, which included the patient’s socio-demographic factors, clinical parameters, diagnoses and treatments at both hospital admission and discharge, clinical events during hospitalization, and outcome. The Ethical Committees of all the participating institutions approved the study. The study was conducted according to the guidelines of the Declaration of Helsinki, and since this study is an observational study, a Clinical Trial Number is not applicable. For more protocol information see The RE.PO.SI study [6].

In this study, the following information were used:

1. The *International Classification of Diseases, Ninth Revision, Clinical Modification* (ICD-9-CM) was employed to categorize diseases [7] and the *Anatomical Therapeutic Chemical Classification System* (ATC) was employed to categorize drugs [8].
2. The severity of chronic conditions affecting the participant was rated using the C*umulative Illness Rating Scale* (CIRS) [9], which includes 14 organs and systems that can be affected by diseases and records the severity of the diseases. CIRS is scored ranging from 0 (“no problem affecting the system) to 4 (“Extremely severe problem and/or immediate treatment required and/or organ failure and/ or severe functional impairment”). In addition, the clinicians were allowed to specify directly the diseases affecting each system using free text. The* Cumulative Illness Rating Scale – Severity Index* (CIRS-SI) result is from the average of the scores in the first 13 categories (excluding the psychiatric/behavioural pathology category). The *Cumulative Illness Rating Scale – Comorbidity Index* (CIRS-CI), represents the number of categories in which a score greater than or equal to 3 was obtained, these scores were used to summarise the burden of chronic conditions affecting the participant.
3. The *Barthel Index* (BI) [10] was used to evaluate disability and physical function in activities of daily living (ADL).
4. Demographics, information on smoking habit and alcohol consumption, as well as civil status, living situation, and education were also collected by the attending physician by interviewing the participant or her/his proxies.

We defined our exposure as follows:

Cognitive impairment was defined as the presence of at least one among the following criteria:

1. A Short Blessed Test (SBT) score of 10 or higher. The SBT was administered during hospitalization when the attending physician deemed the patient stable from the acute condition. The SBT has demonstrated excellent reliability and the ability to accurately discriminate between different levels of cognitive impairment. A score of 10 or higher is consistent with a diagnosis of dementia [11–13]
2. A diagnosis of dementia recorded on the discharge form using ICD-9CM codes (290.0,290.1,290.2,290.3,290.4,291.2,294.1,294.2)
3. A diagnosis of depression noted in the written specifications of the Cumulative Illness Rating Scale (CIRS) by identifying the term "depress*"
4. The chronic prescription of drugs with ATC code”N06D*”
5. An inability to complete the Geriatric Depression Scale-4 (GDS-4) [14] due to cognitive impairment, as registered by the attending physician

Depression was defined as the presence of at least one among the following criteria:

1. *The Geriatric Depression Scale-4* (GDS-4) [14] a score of 2 or higher indicated depression. GDS-4 has been proposed for the screening of depression in older adults and it can be used in hospital and community settings [15] with a sensitivity of 0.77 and a specificity of 0.75 [14].
2. A diagnosis of dementia recorded on the discharge form using ICD-9CM codes (290.0,290.1,290.2,290.3,290.4,291.2,294.1,294.2)
3. A diagnosis of depression noted in the written specifications of the Cumulative Illness Rating Scale (CIRS) by identifying the term "depress*"
4. The chronic prescription of drugs with ATC code”N06A*”

The study population was further stratified in four mutually exclusive categories: 1) No cognitive impairment/ no depression; 2) Depression alone; 3) Cognitive impairment alone; 4) Cognitive impairment and depression.

Mortality data was collected through phone interviews conducted at predefined follow-up intervals (3 and 12 months), during which information about the date of death was obtained from a proxy of the patient. For this study, we focused on 6-month mortality data. Patients who were still alive at the 3-month follow-up but did not complete the 12-month follow-up were excluded from the study.

### Statistical analysis

The characteristics of the study population were described using counts and proportions, means and standard deviations, or medians and interquartile ranges (IQR), as appropriate. Differences between groups were assessed using Chi-squared tests, Fisher’s exact test, or Mann–Whitney U-test, as appropriate. Additionally, the Shapiro–Wilk test was employed to assess the normality of continuous variables to determine the suitability of parametric or non-parametric tests for further analysis. The association between the presence of cognitive impairment and/or depression and 6-month mortality was investigated using Cox proportional hazards models. The proportionality of hazards was evaluated through graphical inspection of Schoenfeld residuals. All analyses were conducted using R version 4.3.0 (R Foundation for Statistical Computing, Vienna).

## Results

**Fig. 1:**
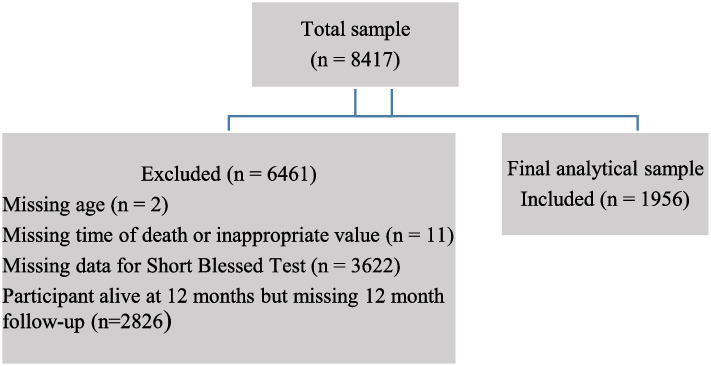
Flow chart for the inclusion of participants in the study

An initial sample of 8417 participants was included in the Re.PO.SI register. Participant with missing information on age or the SBT were excluded (*N* = 3624). Further, 11 participants were excluded because of errors in the date of death. Participants who were not dead within 12 months from hospital admission and missing the 12-month follow-up were excluded (*N* = 2826). The final analytical sample was 1956 participants, depicted in Fig. 1.

As shown in Supplementary Table S1, those included in the study were rather similar to those excluded, although the former were older and prescribed, on average, more medications than the latter. In this secondary analysis, the interviews and data taken from the Re.Po.SI study [6], yielded a sample of 1956 participants had a median age of 80.0 years (IQR: 73.0—85.0) and half of the population was male (49.4%; *N* = 967), as shown in Table 1. The median number of years of education was 5 (IQR: 5–8) and nearly half of the participants were married (52.7%, *N* = 1001). The median score on the BI was 91.0 (IQR: 68.0—100.0). The median number of prescribed medications was 6.0 (IQR: 4.0—8.0) while the median CIRS-CI was 3.0 (IQR: 2.0—4.0). In total, 44.9% and 46.2% of the study population meet the criteria for cognitive impairment or depression, respectively. When the study population was stratified in four mutually exclusive categories, a third of the population was found to be free from both conditions, 24.3% were affected by both conditions simultaneously, 20.7% were affected by cognitive impairment only and 21.9% were affected by depression only. The 6-month mortality was nearly 25% (*N* = 484) and the median follow-up was 71.5 days.

**Table 1:** Demographics and characteristics of participants stratified by 6-month mortality

	**Final analytical sample**	**Death at 6 months = NO**	**Death at 6 months = YES**	***p***
**Number of participants,** n (%)	**1956 (100)**	1472 (75.3)	484 (24.7)	
**Age,** median [IQR]	**80.0 [73.0, 85.0]**	79.0 [73.0, 84.0]	82.0 [76.0, 87.0]	< 0.001^c^
**Sex** = **Male** (%)	**967 (49.4)**	695 (47.2)	272 (56.2)	0.001^a^
**Education years,** median [IQR]	**5.0 [5.0, 8.0]**	5.0 [5.0, 8.0]	5.0 [5.0, 8.0]	0.958^c^
**Civil status,** n (%)				0.642^a^
* married*	**1001 (52.7)**	757 (52.8)	244 (52.5)	
* widowed*	**720 (37.9)**	549 (38.3)	171 (36.8)	
* separated*	**29 (1.5)**	21 (1.5)	8 (1.7)	
* divorced*	**32 (1.7)**	25 (1.7)	7 (1.5)	
* single*	**116 (6.1)**	81 (5.7)	35 (7.5)	
**Living with = others** (%)	**1394 (75.1)**	1050 (74.5)	344 (76.8)	0.367^a^
**Alcoho**l **consumption** (%)				0.055^a^
* Never*	**1013 (53.1)**	755 (52.5)	258 (55.1)	
* ex-drinker*	**103 (5.4)**	69 (4.8)	34 (7.3)	
* drinker*	**318 (16.7)**	253 (17.6)	65 (13.9)	
* Social drinker*	**472 (24.8)**	361 (25.1)	111 (23.7)	
**Collar**^**1**^** = White** (%)	**549 (38.2)**	407 (37.6)	142 (40.1)	0.431^a^
**P-ADL**^**2**^ median [IQR]	**91.0 [68.0, 100.0]**	93.0 [74.0, 100.0]	82.0 [44.2, 96.0]	< 0.001^c^
**Specific P-ADL**^**2**^**: Personal hygiene** (%)				< 0.001^a^
* 0*	**135 (7.0)**	80 (5.5)	55 (11.5)	
* 1*	**102 (5.3)**	51 (3.5)	51 (10.6)	
* 3*	**229 (11.8)**	155 (10.6)	74 (15.4)	
* 4*	**277 (14.3)**	203 (13.9)	74 (15.4)	
* 5*	**1192 (61.6)**	967 (66.4)	225 (47.0)	
**Specific P-ADL**^**2**^**: Bathing** (%)				< 0.001^a^
* 0*	**211 (10.9)**	131 (9.0)	80 (16.7)	
* 1*	**128 (6.6)**	76 (5.2)	52 (10.9)	
* 3*	**288 (14.9)**	206 (14.1)	82 (17.1)	
* 4*	**318 (16.4)**	221 (15.2)	97 (20.3)	
* 5*	**990 (51.2)**	822 (56.5)	168 (35.1)	
**Specific P-ADL**^**2**^**: Feeding** (%)				< 0.001^a^
* 0*	**65 (3.4)**	41 (2.8)	24 (5.0)	
* 2*	**58 (3.0)**	34 (2.3)	24 (5.0)	
* 5*	**169 (8.7)**	107 (7.3)	62 (12.9)	
* 8*	**211 (10.9)**	144 (9.9)	67 (14.0)	
* 10*	**1432 (74.0)**	1130 (77.6)	302 (63.0)	
**Specific P-ADL**^**2**^**: Toileting** (%)				< 0.001^a^
* 0*	**147 (7.6)**	85 (5.8)	62 (12.9)	
* 2*	**111 (5.7)**	60 (4.1)	51 (10.6)	
* 5*	**160 (8.3)**	117 (8.0)	43 (9.0)	
* 8*	**251 (13.0)**	183 (12.6)	68 (14.2)	
* 10*	**1266 (65.4)**	1011 (69.4)	255 (53.2)	
**Specific P-ADL**^**2**^**: Stair Climbing** (%)				< 0.001^a^
* 0*	**328 (17.0)**	195 (13.4)	133 (27.8)	
* 2*	**150 (7.8)**	106 (7.3)	44 (9.2)	
* 5*	**317 (16.4)**	234 (16.1)	83 (17.3)	
* 8*	**351 (18.1)**	262 (18.0)	89 (18.6)	
* 10*	**789 (40.8)**	659 (45.3)	130 (27.1)	
**Specific P-ADL**^**2**^**: Dressing** (%)				< 0.001^a^
* 0*	**141 (7.3)**	77 (5.3)	64 (13.4)	
* 2*	**123 (6.4)**	70 (4.8)	53 (11.1)	
* 5*	**231 (11.9)**	169 (11.6)	62 (12.9)	
* 8*	**287 (14.8)**	211 (14.5)	76 (15.9)	
* 10*	**1153 (59.6)**	929 (63.8)	224 (46.8)	
**Specific P-ADL**^**2**^**: Bladder control** (%)				< 0.001^a^
* 0*	**152 (7.9)**	98 (6.7)	54 (11.3)	
* 2*	**87 (4.5)**	45 (3.1)	42 (8.8)	
* 5*	**174 (9.0)**	124 (8.5)	50 (10.5)	
* 8*	**285 (14.7)**	205 (14.1)	80 (16.7)	
* 10*	**1236 (63.9)**	984 (67.6)	252 (52.7)	
**Specific P-ADL**^**2**^**: Bowel control** (%)				< 0.001
* 0*	**112 (5.8)**	66 (4.5)	46 (9.6)	
* 2*	**73 (3.8)**	40 (2.7)	33 (6.9)	
* 5*	**133 (6.9)**	87 (6.0)	46 (9.6)	
* 8*	**187 (9.7)**	128 (8.8)	59 (12.3)	
* 10*	**1430 (73.9)**	1135 (78.0)	295 (61.6)	
**Specific P-ADL**^**2**^: **Ambulation** (%)				< 0.001^a^
* 0*	**146 (7.7)**	80 (5.6)	66 (14.2)	
* 3*	**135 (7.1)**	79 (5.5)	56 (12.0)	
* 8*	**285 (15.1)**	211 (14.8)	74 (15.9)	
* 12*	**452 (23.9)**	337 (23.6)	115 (24.7)	
* 15*	**874 (46.2)**	719 (50.4)	155 (33.3)	
**Specific P-ADL**^**2**^**: Wheelchair** (%)				0.019^a^
* 0*	**80 (44.2)**	57 (42.5)	23 (48.9)	
* 1*	**21 (11.6)**	12 (9.0)	9 (19.1)	
* 3*	**20 (11.0)**	16 (11.9)	4 (8.5)	
* 4*	**7 (3.9)**	3 (2.2)	4 (8.5)	
* 5*	**53 (29.3)**	46 (34.3)	7 (14.9)	
**Specific P-ADL**^**2**^**: Bed to chair transfer** (%)				< 0.001^a^
* 0*	**143 (7.4)**	85 (5.8)	58 (12.1)	
* 3*	**152 (7.9)**	96 (6.6)	56 (11.7)	
* 8*	**238 (12.3)**	163 (11.2)	75 (15.7)	
* 12*	**358 (18.5)**	265 (18.2)	93 (19.4)	
* 15*	**1043 (53.9)**	846 (58.1)	197 (41.1)	
**CIRS**^**3**^** Illness Severity Index,** median [IQR]	**1.7 [1.5, 1.9]**	1.6 [1.4, 1.8]	1.8 [1.5, 2.0]	< 0.001^c^
**CIRS**^**3**^** Co-morbidity Index, **^**median**^ [IQR]	**3.0 [2.0, 4.0]**	3.0 [2.0, 4.0]	3.0 [2.0, 5.0]	< 0.001^c^
**Prescribed pharmaceuticals** median [IQR]	**6.0 [4.0, 8.0]**	5.0 [4.0, 8.0]	6.0 [4.0, 8.0]	0.043^c^
**GDS**^**4**^**,** median [IQR]	**1.0 [0.0, 2.0]**	1.0 [0.0, 2.0]	1.0 [1.0, 3.0]	< 0.001^c^
**SBT**^**5**^**,** median [IQR]	**8.0 [3.8, 14.0]**	7.0 [2.0, 13.0]	10.0 [4.0, 19.0]	< 0.001^c^
**Cognitive impairment (CI) = yes** (%)	**879 (44.9)**	608 (41.3)	271 (56.0)	< 0.001^a^
**CIRS ICD for CD**^**6,7**^**,** (%)	**165 (8.4)**	94 (6.4)	71 (14.7)	< 0.001^a^
**CIRS text,** (%)	**162 (8.3)**	92 (6.2)	70 (14.5)	< 0.001^a^
**Pharmaceuticals ATC code for CD**^**8**^**,** (%)	**20 (1.0)**	17 (1.2)	3 (0.6)	0.452^b^
**SBT ≥ 10,** (%)	**860 (44.0)**	593 (40.3)	267 (55.2)	< 0.001^a^
**Depression diagnosis = yes** (%)	**903 (46.2)**	669 (45.4)	234 (48.3)	0.290^a^
**CIRS ICD for depression**^**9**^**,** (%)	**77 (3.9)**	60 (4.1)	17 (3.5)	0.676^a^
**CIRS text for depression,** (%)	**205 (10.5)**	153 (10.4)	52 (10.7)	0.895^a^
**Pharmaceuticals ATC code for depression**^**10**^**,** (%)	**331 (16.9)**	255 (17.3)	76 (15.7)	0.457^a^
**GDS ≥ 2,** (%)	**706 (42.0)**	516 (39.8)	190 (49.5)	0.001^a^
**Presence of CI and/or depression (%)**				< 0.001^a^
* none*	**649 (33.2)**	533 (36.2)	116 (24.0)	
* depression only*	**428 (21.9)**	331 (22.5)	97 (20.0)	
* CI only*	**404 (20.7)**	270 (18.3)	134 (27.7)	
* both*	**475 (24.3)**	338 (23.0)	137 (28.3)	
**Presence of FU**^**11**^** at 12 months = 1 (%)**	**1480 (75.7)**	1422 (96.6)	58 (12.0)	< 0.001^a^
**Days of follow-up** median [IQR]	**180.0 [180.0, 180.0]**	180.0 [180.0, 180.0]	54 [25.0, 94.0]	< 0.001^c^

^1^White-collar workers generally perform job duties in an office or other administrative settings

^2^Personal Activities of Daily Living score with Barthel Scale

^3^Cumulative Illness Rating Scale

^4^Geriatric Depression Scale (4-point scale

^5^Short Blessed Test

^6^Cognitive decline

^7^Presence of ICD (The International Classification of Diseases, Ninth Revision, Clinical Modification) codes for dementia in CIRS (290.0, 290.1, 290.2, 290.3, 290.4, 290.8, 290.8, 290.9, 291.2, 294.1)

^8^Presence of medications coded as "N06D" (Anti-dementia drugs) under the Anatomical Therapeutic Chemical (ATC) Classification System

^9^Presence of ICD (The International Classification of Diseases, Ninth Revision, Clinical Modification) codes for depression in CIRS (296.1, 296.3, 296.4, 298.0, 300.4)

^10^Presence of medications coded as "N06A" (Antidepressants) under the Anatomical Therapeutic Chemical (ATC) Classification System

^11^Follow up

MISSING

Civil status, n (%) = 58 (3.0)

Living with, n (%) = 99 (5.1)

Alcohol consumption, n (%) = 50 (2.6)

Collar, n (%) = 519 (26.5)

Specific P-ADL^2^: Personal hygiene, n (%) = 21(1.1)

Specific P-ADL^2^: Bathing, n (%) = 21(1.1)

Specific P-ADL^2^: Feeding, n (%) = 21(1.1)

Specific P-ADL^2^: Toileting, n (%) = 21(1.1)

Specific P-ADL^2^: Stair Climbing, n (%) = 21(1.1)

Specific P-ADL^2^: Dressing, n (%) = 21(1.1)

Specific P-ADL^2^: Bladder control, n (%) = 22(1.1)

Specific P-ADL^2^: Bowel control, n (%) = 21(1.1)

Specific P-ADL^2^: Ambulation, n (%) = 64(3.3)

Specific P-ADL^2^: Wheelchair, n (%) = 1775 (90)

Specific P-ADL^2^: Bed to chair transfer, n (%) = 22(1.1)

Pharmaceuticals ATC code for CD^8^, n (%) = 2(0.1)

Pharmaceuticals ATC code for depression^10^ n (%) = 2(0.1)

GDS ≥ 2, n (%) = 274 (14.0)

STATISTICAL TEST USED

Chi-squared test^a^

Fisher’s exact test^b^

Mann–Whitney U-test^c^

Those who died within 6 months were more likely to be older (82.0 years vs. 79.0 years), male (56.2% vs. 47.2%), had a reduction in the ability to perform ADL, were moderately dependent on others (BI 82.0 vs. 93.0), and were prescribed with higher number of medications (6.0 vs. 5.0). A diagnosis of cognitive impairment was more likely among those dying within 6 months from the hospitalization (56.0% vs 41.3%, *p* < 0.001), although the proportion of persons with depression was similar within the groups (48.3% vs 45.4%, *p* = 0.290).

Table 2 shows the characteristics of the study population stratified according to the presence of cognitive impairment and/or depression. The median age was higher among those with both conditions (83.0 years old) and was lower among those diagnosed with depression only (77.0 years old). The proportion of males was higher among those with neither condition (61.6%) and was lower among those with both (38.3). The median BI score ranged between 100 (independent in ADL, with neither condition present) and 74.0 (moderately dependent in ADL with both cognitive impairment and depression present). Mortality at six months for the population was nearly one in four participants (24.7), and the group with the highest odds of mortality had cognitive impairment only, effecting one in three participants (33.2).

**Table 2:** Demographics and characteristics of participants stratified by presence of cognitive impairment and/or depression

	**Final analytical sample**	**None**	**Depression only**	**Cognitive Impairment (CI)**^**6**^** only**	**Both**	***P*** value
**Number of participants,** n (%)	**1956 (100)**	649 (33.2)	428 (21.9)	404 (20.7)	475 (24.3)	
**Age,** median [IQR]	**80.0 [73.0, 85.0]**	78.0 [72.0, 83.0]	77.0 [71.0, 83.0]	82.0 [76.0, 87.0]	83.0 [77.0, 87.0]	< 0.001^c^
**Sex** = **Male** (%)	**967 (49.4)**	400 (61.6)	192 (44.9)	193 (47.8)	182 (38.3)	< 0.001^a^
**Education years,** median [IQR]	**5.0 [5.0, 8.0]**	8.0 [5.0, 12.0]	8.0 [5.0, 10.0]	5.0 [5.0, 8.0]	5.0 [5.0, 8.0]	< 0.001^c^
**Civil status,** n (%)						< 0.001^a^
* married*	**1001 (52.7)**	372 (59.4)	233 (55.2)	187 (48.1)	209 (45.3)	
* widowed*	**720 (37.9)**	197 (31.5)	139 (32.9)	165 (42.4)	219 (47.5)	
* separated*	**29 (1.5)**	11 (1.8)	9 (2.1)	6 (1.5)	3 (0.7)	
* divorced*	**32 (1.7)**	12 (1.9)	9 (2.1)	6 (1.5)	5 (1.1)	
* single*	**116 (6.1)**	34 (5.4)	32 (7.6)	25 (6.4)	25 (5.4)	
**Living with = others** (%)	**1394 (75.1)**	453 (73.3)	301 (74.3)	286 (74.1)	354 (79.0)	0.166^a^
**Alcoho**l **consumption** (%)						0.013^a^
* Never*	**1013 (53.1)**	322 (51.1)	216 (51.3)	195 (49.5)	280 (60.7)	
* ex-drinker*	**103 (5.4)**	28 (4.4)	25 (5.9)	30 (7.6)	20 (4.3)	
* drinker*	**318 (16.7)**	117 (18.6)	67 (15.9)	73 (18.5)	61 (13.2)	
* Social drinker*	**472 (24.8)**	163 (25.9)	113 (26.8)	96 (24.4)	100 (21.7)	
**Collar**^**1**^** = White** (%)	**549 (38.2)**	215 (45.6)	134 (43.5)	96 (32.7)	104 (28.7)	< 0.001^a^
**P-ADL**^**2**^ median [IQR]	**91.0 [68.0, 100.0]**	100.0 [89.0, 100.0]	92.0 [77.2, 100.0]	82.0 [53.0, 97.0]	74.0 [39.5, 92.0]	< 0.001^c^
**Specific P-ADL**^**2**^**: Personal hygiene** (%)						< 0.001^a^
* 0*	**135 (7.0)**	18 (2.8)	16 (3.8)	39 (9.8)	62 (13.1)	
* 1*	**102 (5.3)**	13 (2.0)	14 (3.3)	26 (6.5)	49 (10.4)	
* 3*	**229 (11.8)**	34 (5.3)	28 (6.6)	77 (19.2)	90 (19.1)	
* 4*	**277 (14.3)**	53 (8.3)	72 (17.1)	67 (16.8)	85 (18.0)	
* 5*	**1192 (61.6)**	523 (81.6)	292 (69.2)	191 (47.8)	186 (39.4)	
**Specific P-ADL**^**2**^**: Bathing** (%)						< 0.001^a^
* 0*	**211 (10.9)**	29 (4.5)	36 (8.5)	56 (14.0)	90 (19.1)	
* 1*	**128 (6.6)**	18 (2.8)	15 (3.6)	32 (8.0)	63 (13.3)	
* 3*	**288 (14.9)**	45 (7.0)	51 (12.1)	88 (22.0)	104 (22.0)	
* 4*	**318 (16.4)**	71 (11.1)	92 (21.8)	76 (19.0)	79 (16.7)	
* 5*	**990 (51.2)**	478 (74.6)	228 (54.0)	148 (37.0)	136 (28.8)	
**Specific P-ADL**^**2**^**: Feeding** (%)						< 0.001^a^
* 0*	**65 (3.4)**	17 (2.7)	7 (1.7)	20 (5.0)	21 (4.4)	
* 2*	**58 (3.0)**	6 (0.9)	3 (0.7)	16 (4.0)	33 (7.0)	
* 5*	**169 (8.7)**	19 (3.0)	22 (5.2)	49 (12.2)	79 (16.7)	
* 8*	**211 (10.9)**	34 (5.3)	39 (9.2)	61 (15.2)	77 (16.3)	
* 10*	**1432 (74.0)**	565 (88.1)	351 (83.2)	254 (63.5)	262 (55.5)	
**Specific P-ADL**^**2**^**: Toileting** (%)						< 0.001^a^
* 0*	**147 (7.6)**	18 (2.8)	18 (4.3)	42 (10.5)	69 (14.6)	
* 2*	**111 (5.7)**	20 (3.1)	15 (3.6)	32 (8.0)	44 (9.3)	
* 5*	**160 (8.3)**	16 (2.5)	30 (7.1)	45 (11.2)	69 (14.6)	
* 8*	**251 (13.0)**	59 (9.2)	54 (12.8)	60 (15.0)	78 (16.5)	
* 10*	**1266 (65.4)**	528 (82.4)	305 (72.3)	221 (55.2)	212 (44.9)	
**Specific P-ADL**^**2**^**: Stair Climbing** (%)						< 0.001^a^
* 0*	**328 (17.0)**	40 (6.2)	60 (14.2)	94 (23.5)	134 (28.4)	
* 2*	**150 (7.8)**	26 (4.1)	28 (6.6)	35 (8.8)	61 (12.9)	
* 5*	**317 (16.4)**	63 (9.8)	70 (16.6)	79 (19.8)	105 (22.2)	
* 8*	**351 (18.1)**	122 (19.0)	83 (19.7)	70 (17.5)	76 (16.1)	
* 10*	**789 (40.8)**	390 (60.8)	181 (42.9)	122 (30.5)	96 (20.3)	
**Specific P-ADL**^**2**^**: Dressing** (%)						< 0.001^a^
* 0*	**141 (7.3)**	20 (3.1)	15 (3.6)	41 (10.2)	65 (13.8)	
* 2*	**123 (6.4)**	14 (2.2)	19 (4.5)	34 (8.5)	56 (11.9)	
* 5*	**231 (11.9)**	33 (5.1)	44 (10.4)	60 (15.0)	94 (19.9)	
* 8*	**287 (14.8)**	61 (9.5)	62 (14.7)	77 (19.2)	87 (18.4)	
* 10*	**1153 (59.6)**	513 (80.0)	282 (66.8)	188 (47.0)	170 (36.0)	
**Specific P-ADL**^**2**^**: Bladder control** (%)						< 0.001^a^
* 0*	**152 (7.9)**	30 (4.7)	15 (3.6)	43 (10.8)	64 (13.6)	
* 2*	**87 (4.5)**	10 (1.6)	11 (2.6)	25 (6.2)	41 (8.7)	
* 5*	**174 (9.0)**	18 (2.8)	32 (7.6)	54 (13.5)	70 (14.9)	
* 8*	**285 (14.7)**	52 (8.1)	61 (14.5)	77 (19.2)	95 (20.2)	
* 10*	**1236 (63.9)**	531 (82.8)	303 (71.8)	201 (50.2)	201 (42.7)	
**Specific P-ADL**^**2**^**: Bowel control** (%)						< 0.001^a^
* 0*	**112 (5.8)**	19 (3.0)	9 (2.1)	34 (8.5)	50 (10.6)	
* 2*	**73 (3.8)**	11 (1.7)	10 (2.4)	20 (5.0)	32 (6.8)	
* 5*	**133 (6.9)**	17 (2.7)	25 (5.9)	39 (9.8)	52 (11.0)	
* 8*	**187 (9.7)**	26 (4.1)	36 (8.5)	58 (14.5)	67 (14.2)	
* 10*	**1430 (73.9)**	568 (88.6)	342 (81.0)	249 (62.3)	271 (57.4)	
**Specific P-ADL**^**2**^: **Ambulation** (%)						< 0.001^a^
* 0*	**146 (7.7)**	21 (3.3)	21 (5.1)	43 (10.9)	61 (13.5)	
* 3*	**135 (7.1)**	21 (3.3)	23 (5.6)	30 (7.6)	61 (13.5)	
* 8*	**285 (15.1)**	45 (7.1)	62 (15.0)	80 (20.4)	98 (21.7)	
* 12*	**452 (23.9)**	125 (19.7)	96 (23.2)	114 (29.0)	117 (25.9)	
* 15*	**874 (46.2)**	421 (66.5)	212 (51.2)	126 (32.1)	115 (25.4)	
**Specific P-ADL**^**2**^**: Wheelchair** (%)						< 0.001^a^
* 0*	**80 (44.2)**	20 (40.8)	20 (51.3)	17 (47.2)	23 (40.4)	
* 1*	**21 (11.6)**	2 (4.1)	4 (10.3)	4 (11.1)	11 (19.3)	
* 3*	**20 (11.0)**	1 (2.0)	1 (2.6)	8 (22.2)	10 (17.5)	
* 4*	**7 (3.9)**	1 (2.0)	2 (5.1)	3 (8.3)	1 (1.8)	
* 5*	**53 (29.3)**	25 (51.0)	12 (30.8)	4 (11.1)	12 (21.1)	
**Specific P-ADL**^**2**^**: Bed to chair transfer** (%)						< 0.001^a^
* 0*	**143 (7.4)**	19 (3.0)	16 (3.8)	36 (9.0)	72 (15.3)	
* 3*	**152 (7.9)**	28 (4.4)	27 (6.4)	38 (9.5)	59 (12.5)	
* 8*	**238 (12.3)**	33 (5.1)	46 (10.9)	72 (18.0)	87 (18.4)	
* 12*	**358 (18.5)**	90 (14.0)	84 (19.9)	89 (22.3)	95 (20.1)	
* 15*	**1043 (53.9)**	471 (73.5)	249 (59.0)	164 (41.1)	159 (33.7)	
**CIRS**^**3**^** Illness Severity Index,** median [IQR]	**1.7 [1.5, 1.9]**	1.6 [1.4, 1.8]	1.7 [1.5, 1.9]	1.7 [1.5, 1.9]	1.7 [1.5, 2.0]	< 0.001^c^
**CIRS**^**3**^** Co-morbidity Index,** median [IQR]	**3.0 [2.0, 4.0]**	3.0 [1.0, 4.0]	3.0 [2.0, 4.0]	3.0 [2.0, 4.0]	3.0 [2.0, 5.0]	< 0.001^c^
**Prescribed pharmaceuticals** median [IQR]	**6.0 [4.0, 8.0]**	5.0 [3.0, 7.0]	6.0 [4.0, 8.0]	5.0 [3.0, 8.0]	6.0 [4.0, 8.0]	< 0.001^c^
**GDS**^**4**^**,** median [IQR]	**1.0 [0.0, 2.0]**	0.0 [0.0, 1.0]	2.0 [2.0, 3.0]	1.0 [0.0, 1.0]	2.0 [2.0, 3.0]	< 0.001^c^
**SBT**^**5**^**,** median [IQR]	**8.0 [3.8, 14.0]**	4.0 [0.0, 6.0]	4.0 [2.0, 6.0]	15.0 [12.0, 21.0]	16.0 [12.0, 22.0]	< 0.001^c^
**Cognitive impairment diagnosis = yes** (%)	**879 (44.9)**	0 (0.0)	0 (0.0)	404 (100.0)	475 (100.0)	< 0.001^a^
**CIRS ICD for CI**^**6,7**^**,** (%)	**165 (8.4)**	0 (0.0)	0 (0.0)	68 (16.8)	97 (20.4)	< 0.001^a^
**CIRS text,** (%)	**162 (8.3)**	0 (0.0)	0 (0.0)	66 (16.3)	96 (20.2)	< 0.001^a^
**Pharmaceuticals ATC code for CI**^**8**^**,** (%)	**20 (1.0)**	0 ( 0.0)	0 ( 0.0)	4 (1.0)	16 ( 3.4)	< 0.001^b^
**SBT ≥ 10,** (%)	**860 (44.0)**	0 (0.0)	0 (0.0)	397 (98.3)	463 (97.5)	< 0.001^a^
**Depression diagnosis = yes** (%)	**903 (46.2)**	0 (0.0)	428 (100.0)	0 ( 0.0)	475 (100.0)	< 0.001^a^
**CIRS ICD for depression**^**9**^**,** (%)	**77 (3.9)**	0 (0.0)	38 ( 8.9)	0 ( 0.0)	39 ( 8.2)	< 0.001^a^
**CIRS text for depression,** (%)	**205 (10.5)**	0 ( 0.0)	104 (24.3)	0 ( 0.0)	101 (21.3)	< 0.001^a^
**Pharmaceuticals ATC code for depression**^**10**^**,** (%)	**331 (16.9)**	0 ( 0.0)	147 (34.4)	0 ( 0.0)	184 (38.8)	< 0.001^a^
**GDS ≥ 2,** (%)	**706 (42.0)**	0 ( 0.0)	340 (83.5)	0 ( 0.0)	366 (85.9)	< 0.001^a^
**Death at 6 months = yes (%)**	**484 (24.7)**	116 ( 17.9)	97 ( 22.7)	134 ( 33.2)	137 ( 28.8)	< 0.001^a^
**Days of follow up** median [IQR]	**180.0 [180.0, 180.0]**	180.0 [180.0, 180.0]	180.0 [180.0, 180.0]	180.0 [91.0, 180.0]	180.0 [115.0, 180.0]	0.061

^1^White-collar workers generally perform job duties in an office or other administrative settings

^2^Personal Activities of Daily Living score with Barthel Scale

^3^Cumulative Illness Rating Scale

^4^Geriatric Depression Scale (4-point scale)

^5^Short Blessed Test

^6^Cognitive impairment

^7^Presence of ICD (The International Classification of Diseases, Ninth Revision, Clinical Modification) codes for dementia in CIRS (290.0, 290.1, 290.2, 290.3, 290.4, 290.8, 290.8, 290.9, 291.2, 294.1)

^8^Presence of medications coded as "N06D" (Anti-dementia drugs) under the Anatomical Therapeutic Chemical (ATC) Classification System

^9^Presence of ICD (The International Classification of Diseases, Ninth Revision, Clinical Modification) codes for depression in CIRS (296.1, 296.3, 296.4, 298.0, 300.4)

^10^Presence of medications coded as "N06A" (Antidepressants) under the Anatomical Therapeutic Chemical (ATC) Classification System

^11^Follow up

MISSING

Civil status, n (%) = 58 (3.0)

Living with, n (%) = 99 (5.1)

Alcohol consumption, n (%) = 50 (2.6)

Collar, n (%) = 519 (26.5)

Specific P-ADL^2^: Personal hygiene, n (%) = 21(1.1)

Specific P-ADL^2^: Bathing, n (%) = 21(1.1)

Specific P-ADL^2^: Feeding, n (%) = 21(1.1)

Specific P-ADL^2^: Toileting, n (%) = 21(1.1)

Specific P-ADL^2^: Stair Climbing, n (%) = 21(1.1)

Specific P-ADL^2^: Dressing, n (%) = 21(1.1)

Specific P-ADL^2^: Bladder control, n (%) = 22(1.1)

Specific P-ADL^2^: Bowel control, n (%) = 21(1.1)

Specific P-ADL^2^: Ambulation, n (%) = 64(3.3)

Specific P-ADL^2^: Wheelchair, n (%) = 1775 (90)

Specific P-ADL^2^: Bed to chair transfer, n (%) = 22(1.1)

Pharmaceuticals ATC code for CD^8^, n (%) = 2(0.1)

Pharmaceuticals ATC code for depression^10^ n (%) = 2(0.1)

GDS ≥ 2, n (%) = 274 (14.0)

STATISTICAL TEST USED

Chi-squared test^a^

Fisher’s exact test^b^

Mann–Whitney U-test^c^

As shown in Table 3, the group with the highest incidence rate of death (IR, per 100 person-years) was cognitive impairment alone (IR 81.1, CI 57.9–115.0), followed by cognitive impairment with depression (IR 68.9, CI 49.2–97.3).

**Table 3:** Incidence rates (IR) of death (per 100 people/year) for variable with mutually exclusive classes between cognitive impairment and depression

	**Unweighted IR (95% CI)**	**Weighted IR (95% CI)**
**No cognitive impairment, no depression**	**43.4 (29.9–62.7)**	41.1 (28.6 – 59.2)
**No cognitive impairment, depression**	**55.9 (37.1 – 84.6)**	54.0 (36.5 – 80.3)
**Cognitive impairment, no depression**	**81.1 (57.9 – 115.0)**	89.0 (65.0 – 123.0)
**Cognitive impairment, depression**	**68.9 (49.2 – 97.3)**	73.9 (53.8 – 102.0)

The weighted incidence rates (IR) and their confidence intervals (CI) was calculated by dividing the age variable into quintiles. This approach ensures that the age distribution was evenly represented across different groups, providing a more accurate comparison of IR

In Table 4, the groups with cognitive impairment and/or depression were compared to those with neither condition. People with cognitive impairment only exhibited the highest increase in the relative hazard of 6-month mortality (Hazard Ratio [HR]: 2.1; 95% CI: 1.6–2.7), followed by those with both conditions (HR: 1.7; 95% CI: 1.4–2.2), and finally those affected by depression only (HR: 1.3; 95% CI: 1.0–1.81.7). These results were confirmed in the adjusted analysis, though slightly attenuated.

**Table 4:** Hazard ratio (HR) for association between 6-month mortality and a variable with 4 mutually exclusive classes between cognitive impairment and depression

	**HR (95% CI) unadjusted**	**HR (95% CI) adjusted**^a^
**No cognitive impairment, no depression**	**REF**	REF
**No cognitive impairment, depression**	**1.30 (1.00 – 1.71)**	1.37(1.03 – 1.84)
**Cognitive impairment, no depression**	**2.08 (1.63 – 2.67)**	1.94 (1.47 – 2.56)
**Cognitive impairment, depression**	**1.75 (1.36 – 2.24)**	1.74 (1.33 – 2.29)

^a^Adjusted for age, sex, years of education, prescribed pharmaceuticals and civil status

We also conducted a sensitivity analysis including cognitive decline, depression, and their multiplicative interaction term, shown in Supplementary Table S2, confirming the strong association between cognitive decline and mortality, the association between depression and mortality and the presence of a negative interaction between cognitive decline and depression.

Lastly, we conducted a sensitivity analysis including all study participants and censoring them at the date of the last available follow-up. The results were similar to those shown in the main analyses, for both direction and magnitude shown in Supplementary Table S3.

## Discussion

In this secondary analysis of the RE.PO.SI data, we identified that both cognitive impairment and depression are prevalent in a population of older persons acutely admitted to the hospital, since nearly a quarter (24%) of our study population was affected by both conditions. The cumulative mortality at six month was almost 25% and, although those with both cognitive impairment and depression were older, exhibited greater dependence in their ADLs, had a higher burden of chronic illnesses, they exhibited a risk of dying similar to those with cognitive impairment only. Lastly, in the depression only group there was a lower risk of dying at six months, compared to both groups with cognitive impairment and cognitive impairment with depression.

In this study, roughly 22% of the study participants had depression only. These findings are similar to other studies, confirming that depression is common in older physically ill patients in general hospitals with a mean prevalence of 29% [16]. Similarly, we identified that nearly 21% of the study participants had a cognitive impairment which concurred with a recent cohort study of more than 21,000 hospitalised older adults, of which 27% had a cognitive impairment [17]. Furthermore, our study results correspond with earlier findings that cognitive impairment is independently associated with mortality [17]. In our analysis, individuals with depression only showed a lower six-month mortality risk compared to those with cognitive impairment, with or without depression, in both the unadjusted and adjusted models. However, in the adjusted Cox proportional hazards model, there was no statistically significant difference (adjusted HR 1.33, 95% CI: 0.99–1.79). This suggests a possible trend of higher mortality in groups with cognitive impairment, regardless of the presence of depression. This trend is evident in Table 3, where the age-adjusted incidence rate is lowest in the group without cognitive impairment or depression and highest in the group with cognitive impairment only; with intermediate values in the other two groups. Nonetheless, the overlap of the 95% confidence intervals indicates that these results should be interpreted with caution, highlighting the need for further studies to validate these observations.

This finding contradicts previous research from a meta-study reported that depression is associated with an increased risk of all‐cause mortality [18]. It also contradicts several earlier studies that concluded that when depression is added to cognitive impairment, mortality increases [19, 20], as we found no increase of mortality in our study. While our study refutes multiple earlier findings, it concurs with a recent study published exploring cognitive impairment and depression, in older people hospitalized for hip fractures, as their study too identified that people with depression only had a lower risk of mortality after discharge [21]. Nonetheless, the overlap of the 95% confidence intervals indicates that these results should be interpreted with caution, highlighting the need for further studies to validate these observations.

While the participants in the RE.PO.SI study received a through medication reviews in an effort to optimize treatment [5], this effort may have fallen short related to the use of anti-depressants in our cohort, since the older people in our study population were under prescribed depression medications despite having a depression diagnosis. Previously reported findings in the literature have identified that very few older adults admitted to acute hospitals are diagnosed with depression during their inpatient stays, and that opportunities for improving the mental health as well as physical health appear to be often missed in this population [22].

Merely 17% of our participants were prescribed antidepressant medications, despite nearly 46% identified as having some level of depression (dependent on which depression scale or instrument was used). Despite this large gap, our study had similar antidepressant prescription rates similar to a comprehensive geriatric assessment study of older inpatient people in Sweden, where roughly 60% of the population had some level of depression, but only 30% were prescribed anti-depressants [23]. Prescribing patterns of anti-depressants may vary, and it is possible that some clinicians despite identifying depression, may decline to prescribe anti-depressants to their older patients [24]. This could be due to some uncertainty related to questions of efficacy, or possible concerns related to other comorbidities and medications. Furthermore, there is no way to know if some of the older adults with a depression diagnosis in our study may have been reluctant to seek care or treatment. Furthermore, previously reported findings in the literature have identified that very few older adults admitted to acute hospitals are diagnosed with depression during their inpatient stays, and that opportunities for improving the mental health as well as physical health appear to be often missed [22].

Older people experiencing disorders such as depression and cognitive impairment are at a higher risk of experiencing poor quality of life, disability, increased risk for somatic disorders, and increased mortality [25]. Despite these facts, our study suggests that depression did not increase the risk for mortality. One possible interpretation is the protective impact of antidepressant therapy on mortality previously reported in the literature [26], however it should be noted that the exclusion of participants on antidepressant therapy from our analysis did not change the outcome. It is also possible that doctors were more likely to prescribe antidepressant medication to those people who were identified as at lower risk of short-term mortality, regardless of cognitive impairment, promoting a “reverse causality”. The difficult differential diagnosis between depression and cognitive impairment could further complicate the interpretation of the results. Our results highlight the complexity of the relationship between mortality, depression and cognitive impairment, suggesting the need to standardize the diagnostic methodologies for the classification of these pathologies and emphasizing the need for a holistic management of patients with these conditions.

Future studies are warranted to further explore and untangle the association of depression, cognitive impairment and mortality in older people. Such a study designed should include previous mental health history, including levels of depression and cognitive impairment, medications, therapies and ADL status, prior to, during and after hospital care to better comprehend this phenomenon.

Our study has both strengths and weaknesses. On the positive side, this study’s focus was on older people requiring unplanned hospital admissions, which is an under-researched group, especially when it comes to exploring their mental health. Using the Re.Po.SI data gave us a large sample size and diverse populations, since it represents all territories of Italy. Lastly, we used multiple sources to identify both depression and cognitive impairments, which allowed more diversity in the data. Some weaknesses are our study lacks data on the participant’s mental health history and duration of current depressive symptoms; it was not possible to determine whether depression was a reaction to the stress associated with acute illness and hospital admission. Additionally, we analysed antidepressants as a single class rather than as individual drugs: this approach may have masked specific effects associated with different types of antidepressants. Lastly, the sample methodology may have had falsely inflated the mortality rate. However, the sensitivity analysis conducted showed results similar to those obtained from the main analyses.

The stress of getting sick and being hospitalized can be considered an adverse condition for older people and this increases the risk of depression [27]. Due to the design of this study, we had no way of knowing what attitudes people had about their mental health conditions, and if they had negative views about receiving anti-depression treatment.

Another methodological weakness to the study is the use of different depression scales and instruments, which did not always show a statistically significant relationship to a diagnosis of depression. For example, CIRS has good validity and interrater reliability [28], and maybe useful in developing differential illness profiles associated with mortality, hospitalization, and disability. However, the data used is from self- and physician-report surveys, with archival data drawn from medical charts and facility records [29], could be subjective. Similarly while the GDS-4 [14] is one of many scales that can be used for screening depression in older adults, this scale’s strength is that it is considered easier to use in people with cognitive impairment because of the simple responses (yes–no), and it can be used in hospital and community settings [15]. However, the GDS-4’s clinical value is limited in assessing the severity of a person’s depressive episode [14], and nearly a third of our participants had missing data for this screen. It is prudent to be mindful that depression-screening tools are only tools. Clinicians need to use person-centered care [30] approaches with personal interviews to truly understand the person behind the patient with cognitive impairment with depression, so the best clinical analysis and diagnosis can be made.

## Conclusion

The risk of dying within 6 month from an acute hospitalization was similar for both older people with cognitive impairment and for those with cognitive impairment and depression. While depression alone may contribute to mortality risk, cognitive impairment appears to play a more substantial role in increasing this risk, even though older adults with depression were generally under-prescribed antidepressants. Further research is needed to confirm these findings and explore potential underlying mechanisms.

## Supplementary Information

Supplementary Material 1.

## Data Availability

The datasets used and analysed during the current study using data and materials from Re.Po.SI are available from the corresponding authors on reasonable request. This article is licensed under a Creative Commons Attribution 4.0 International License, which permits use, sharing, adaptation, distribution and reproduction in any medium or format, as long as you give appropriate credit to the original author(s) and the source, provide a link to the Creative Commons licence, and indicate if changes were made.

## Appendix

Investigators and co-authors of the REPOSI (REgistro POliterapie SIMI, Società Italiana di Medicina Interna) Study Group are as follows:

***Steering Committee:*** Pier Mannuccio Mannucci (Chair) (Fondazione IRCCS Cà Granda Ospedale Maggiore Policlinico, Milano), Alessandro Nobili (co-chair) (Istituto di Ricerche Farmacologiche Mario Negri IRCCS, Milano), Giorgio Sesti (Presidente SIMI), Antonello Pietrangelo (Direttore CRIS – SIMI), Nicola Montano (Fondazione IRCCS Cà Granda Ospedale Maggiore Policlinico, Milano), Antonio De Vincentis (Policlinico Universitario Campus Bio-Medico, Roma), Alessandra Marengoni (Spedali Civili di Brescia, Brescia), Mauro Tettamanti, Luca Pasina, Carlotta Franchi (Istituto di Ricerche Farmacologiche Mario Negri IRCCS, Milano).

***Clinical Data Monitoring and Revision***: Francesca Orsini, Massimo Cartabia, Gabriella Miglio (Istituto di Ricerche Farmacologiche Mario Negri IRCCS, Milano).

***Database Management and Statistics***: Alessia Antonella Galbussera, Ilaria Ardoino, Alessio Novella, Silvia Cantiero, Enrico Nicolis (Istituto di Ricerche Farmacologiche Mario Negri IRCCS, Milano).

***Investigators***:

- Domenico Prisco, Elena Silvestri, Giacomo Emmi, Alessandra Bettiol, Irene Mattioli, Matteo Mazzetti, Edoardo Biancalana (Azienda Ospedaliero Universitaria Careggi Firenze, SOD Medicina Interna Interdisciplinare);
- Gianni Biolo, Michela Zanetti, Giacomo Bartelloni, Michele Zaccari, Massimiliano Chiuch, Ilaria Martini (Azienda Sanitaria Universitaria Integrata di Trieste, Clinica Medica Generale e Terapia Medica);
- Matteo Pirro, Graziana Lupattelli, Vanessa Bianconi, Riccardo Alcidi, Alessia Giotta, Massimo R. Mannarino (Azienda Ospedaliera Santa Maria della Misericordia, Perugia, Medicina Interna, Angiologia Malattie da Arteriosclerosi);
- Domenico Girelli, Fabiana Busti, Giacomo Marchi (Azienda Ospedaliera Universitaria Integrata di Verona, Verona, Medicina d’Urgenza);
- Nicola Veronese, Mario Barbagallo, Ligia Dominguez, Vincenza Beneduce, Federica Cacioppo, Stefano Ciriminna, Andrea Asaro, Chiara Giannettino, Claudia Canizzo, Giovanna Ottavia Plano, Anna Fazzari (Azienda Ospedaliera Universitaria Policlinico Giaccone Policlinico di Palermo, Palermo, Unità Operativa di Geriatria e Lungodegenza);
- Salvatore Corrao, Giuseppe Natoli, Salvatore Mularo, Massimo Raspanti, Christiano Argano, Federica Cavallaro, Valentina Orlando (A.R.N.A.S. Civico, Di Cristina, Benfratelli, Palermo, UOC Medicina Interna ad Indirizzo Geriatrico-Riabilitativo);
- Marco Zoli, Maria Laura Matacena, Giuseppe Orio, Eleonora Magnolfi, Giovanni Serafini, Mattia Brunori, Ilaria Lazzari, Angelo Simili (Azienda Ospedaliera Universitaria Policlinico S. Orsola-Malpighi, Bologna, Unità Operativa di Medicina Interna Zoli);
- Maria Domenica Cappellini, Giovanna Fabio, Margherita Migone De Amicis, Giacomo De Luca, Natalia Scaramellini, Valeria Di Stefano, Simona Leoni, Sonia Seghezzi, Alessandra Danuta Di Mauro, Diletta Maira, Marta Mancarella (Fondazione IRCCS Cà Granda Ospedale Maggiore Policlinico, Milano, Unità Operativa Medicina Interna IA).
- Tiziano Lucchi, Paola Nicolini, Gabriele Ghidini, Miriana Martelengo, Maddalena Fabrizi, Giulia Vigani, Arturo Cerizza, Rita Deda, Miriam Zappa (Fondazione IRCCS Cà Granda Ospedale Maggiore Policlinico, Milano, Geriatria);
- Antonio Di Sabatino, Emanuela Miceli, Marco Vincenzo Lenti, Martina Pisati, Lavinia Pitotti, Valentina Antoci, Ginevra Cambiè, Costanza Caccia Dominioni (IRCCS Policlinico San Matteo di Pavia, Pavia, Clinica Medica I, Reparto 11);
- Roberto Pontremoli, Valentina Beccati, Giulia Nobili, Giovanna Leoncini, Jacopo Alberto, Federico Cattaneo (IRCCS Azienda Ospedaliera Universitaria San Martino-IST di Genova, Genova, Clinica di Medicina Interna 2);
- Francesco Cipollone, Maria Teresa Guagnano, Marco Bucci, Ilaria Rossi, Damiano D’Ardes, Alessia Cipollone, Paola Vizzarri (Ospedale Clinicizzato SS. Annunziata, Chieti, Clinica Medica);
- Gerardo Mancuso, Daniela Calipari, Mosè Bartone (Ospedale Giovanni Paolo II Lamezia Terme, Catanzaro, Unità Operativa Complessa Medicina Interna);
- Roberto Manetti, Marta Chiara Sircana, Maria Berria, Alessandro Delitala, Pierluigi Meloni (Cliniche San Pietro, Azienda Ospedaliera Universitaria di Sassari, S.C. Clinica Medica);
- Maurizio Muscaritoli, Alessio Molfino, Enrico Petrillo, Antonella Giorgi, Christian Gracin, Giovanni Imbimbo, Carmen Gallicchio, Ottavio Martellucci *(Policlinico Umberto I, Sapienza Università di Roma, Medicina Interna e Nutrizione Clinica Policlinico Umberto I);*
- Alessandra Marengoni, Daniela Lucente, Francesca Manzoni, Annalisa Pirozzi, Alberto Zucchelli, Thelma Geneletti, Eleonora Carlotti *(SC Geriatria, Spedali Civili, Montichiari, Brescia);*
- Antonio Picardi, Umberto Vespasiani Gentilucci, Paolo Gallo *(Università Campus Bio-Medico, Roma, Medicina Clinica-Epatologia);*
- Giuseppe Bellelli, Maurizio Corsi, Chukwuma Okoye, Paolo Mazzola, Maria Cristina Ferrara, Alice Ornago, Elena Pinardi, Alberto Finazzi, Lavinia Vitali, Armida Soccali, Alice Rivolta *(Università degli Studi di Milano-Bicocca e Unità Operativa Complessa di Geriatria, IRCCS Fondazione S. Gerardo, Monza);*
- Franco Arturi, Elena Succurro, Melania Melina, Federica Giofrè, Francesca Cosentino, Delia Chiarello, Francesco Andreozzi *(Università degli Studi Magna Graecia, Azienda Ospedaliera-Universitaria “Renato Dulbecco”, Catanzaro, Unità Operativa Complessa di Medicina Interna);*
- Antonio Brucato, Teresa De Falco, Enrica Negro, Martino Brenna, Lucia Trotta, Giovanni Lorenzo Squintani, Giacomo Iacomelli, Giulia Colazzo *(ASST Fatebenefratelli - Sacco, Milano, Medicina Interna);*
- Paolo Simioni, Irene Bertozzi, Sara Angela Malerba, Camilla Portinari, Giordano Antonello, Cecilia Fortino, Maria Luigia Randi* (Azienda Ospedaliera Università di Padova, Padova, Clinica Medica I);*
- Roberto Manfredini, Benedetta Boari, Alfredo De Giorgi, Ruana Tiseo, Giulia Marta Viglione, Caterina Savriè *(Azienda Ospedaliera - Universitaria Sant'Anna, Ferrara, Unità Operativa Clinica Medica);*
- Giuseppe Paolisso, Maria Rosaria Rizzo, Claudia Catalano, Irene Di Meo, Michele Cerasuolo *(Azienda Ospedaliera Universitaria Luigi Vanvitelli- Università della Campania Luigi Vanvitelli, Napoli, Unità Operativa Complessa di Geriatria e Medicina Interna);*
- Claudio Borghi, Federica Piani, Federico Ruscelli, Chiara Baldini, Giulia Boni *(Medicina Interna Cardiovascolare, IRCCS Azienda Ospedaliero-Universitaria di Bologna);*
- Carlo Sabbà, Francesco Saverio Vella, Patrizia Suppressa, Giovanni Michele De Vincenzo, Alessio Comitangelo, Emanuele Amoruso, Carlo Custodero, Giuseppe Re, Chiara Maria Palmisano, Andrea Schilardi *(Azienda Ospedaliero-Universitaria Consorziale Policlinico di Bari, Bari, Medicina Interna Universitaria C. Frugoni);*
- Luigi Fenoglio, Christian Bracco, Giulia Racca, Alessia Valentina Giraudo, Salvatore D’Agnano, Giorgia Sasia, Irene Ruocco *(Azienda Sanitaria Ospedaliera Santa Croce e Carle di Cuneo, Cuneo, S. C. Medicina Interna);*
- Anna Ludovica Fracanzani, Silvia Tiraboschi, Annalisa Cespiati, Giovanna Oberti, Giordano Sigon, Felice Cinque, Lucia Colavolpe, Jaqueline Currà, Francesca Alletto, Natalia Scaramellini, Simona Leoni, Alessandra Danuta Di Mauro, Gianpaolo Benzoni, Margherita Re *(Fondazione IRCCS Cà Granda Ospedale Maggiore Policlinico, Milano, UOC Medicina Generale ad Indirizzo Metabolico);*
- Flora Peyvandi, Raffaella Rossio, Giulia Colombo, Pasquale Agosti, Erica Pagliaro, Eleonora Semproni *(Fondazione IRCCS Cà Granda Ospedale Maggiore Policlinico, Milano, Medicina Interna 2, Ematologia non tumorale e Coagulopatie);*
- Ciro Canetta, Valter Monzani, Valeria Savojardo, Giuliana Ceriani, Christian Folli *(Fondazione IRCCS Cà Granda Ospedale Maggiore Policlinico, Milano, Medicina Interna Alta Intensità di Cure);*
- Fabrizio Montecucco, Cristina Michelauz, Elisa Schiavetta, Chiara Olivero, Simone Isoppo, Curzia Tortorella, Federico Carbone, Luca Liberale *(IRCCS Ospedale Policlinico San Martino e Università di Genova, Genova, Clinica Medica 1, Medicina Interna e Specialità Mediche);*
- Nicola Lucio Liberato, Tiziana Tognin (*ASST di Pavia, UOSD Medicina Interna, Ospedale di Casorate Primo, Pavia);*
- Francesco Purrello, Antonino Di Pino, Salvatore Piro, Maurizio Di Marco *(Ospedale Garibaldi Nesima, Catania, Unità Operativa Complessa di Medicina Interna);*
- Renzo Rozzini, Lina Falanga, Maria Stella Pisciotta, Francesco Baffa Bellucci, Stefano Boffelli, Camillo Ferrandina, Francesca Mazzeo, Elena Spazzini, Giulia Cono, Giulia Cesaroni *(**Ospedale Poliambulanza, Brescia, Medicina Interna e Geriatria);*
- Giuseppe Montrucchio, Paolo Peasso, Edoardo Favale, Cesare Poletto, Carl Margaria, Maura Sanino, Valeria Bianchi, Beatrice Bovo, Beatrice Brusasco, Gabriele Giuliano, Marco Angeleri (*(Dipartimento di Scienze Mediche, Università di Torino,* *Città della Scienza e della Salute, Torino, Medicina Interna 2 Unità Indirizzo d'Urgenza;)*
- Luigina Guasti, Francesca Rotunno, Luana Castiglioni, Andrea Maresca, Alessandro Squizzato, Leonardo Campiotti, Alessandra Grossi, Roberto Davide Diprizio, Francesco Dentali, Veronica Behnke *(Università degli Studi dell'Insubria, Ospedale di Circolo e Fondazione Macchi, Varese, Medicina e Geriatria);*
- Marco Bertolotti, Chiara Mussi, Giulia Lancellotti, Laura Orlandi, Federica Di Zio, Gabriele Luppi Francesca D’Imprima, Chiara Mazza, Claudia Grandi* (Università di Modena e Reggio Emilia, Azienda Ospedaliero-Universitaria di Modena; Ospedale Civile di Baggiovara, Unità Operativa di Geriatria);*
- Angela Sciacqua, Maria Perticone, Raffaele Maio, Aleandra Scozzafava, Valentino Condoleo, Elvira Clausi, Giuseppe Armentaro, Alberto Panza, Alessia Grandinetti, Maria Rosa *(Università Magna Graecia Policlinico Renato Dulbecco, Catanzaro, Unità Operativa Malattie Cardiovascolari Geriatriche);*
- Gianluca Moroncini, Emanuele Filippini, Devis Benfaremo *(Clinica Medica, Azienda Ospedaliera- Universitaria delle Marche, Ancona);*
- Salvatore Minisola, Luciano Colangelo, Mirella Cilli, Giancarlo Labbadia, Jessica Pepe *(Policlinico Umberto I, Roma, SMSC03 - Medicina Interna F e Malattie Metaboliche dell'Osso);*
- Pietro Castellino, Luca Zanoli, Agostino Gaudio, Anastasia Xourafa, Concetta Spichetti, Serena Torre, Alfio Gennaro (*Azienda Ospedaliera Universitaria Policlinico – V. Emanuele, Catania, Dipartimento di Medicina**);*
- Guido Moreo, Silvia Prolo, Gloria Pina, Lorenzo Magni *(Clinica San Carlo Casa di Cura Polispecialistica, Paderno Dugnano, Milano, Unità Operativa di Medicina Generale Emilio Bernardelli);*
- Alberto Ballestrero, Fabio Ferrando, Roberta Gonella, Domenico Cerminara, Paolo Setti, Chiara Traverso, Camilla Scarsi *(Clinica Di Medicina Interna ad Indirizzo Oncologico, Azienda Ospedaliera Università San Martino di Genova);*
- Anna Linda Patti, Giuseppe Famularo, Patrizia Tarsitani, Tiziana Morretti, Andrea Aglitti *(Azienda Ospedaliera San Camillo Forlanini, Roma, Medicina Interna II);*
- Marcello Giuseppe Maggio, Fulvio Lauretani, Marco Salvi, Irene Zucchini, Gian Paolo Ceda, Simonetta Morganti, Andrea Artoni, Margherita Grossi, Chiara Cattabiani, Elena Bronzoni, Aida Hoxha, Beatrice Tanzi *(Azienda Ospedaliero Universitaria di Parma, U.O.C Clinica Geriatrica);*
- Stefano Del Giacco, Davide Firinu, Giulia Costanzo, Andrea Giovanni Ledda, Salvatore Chessa *(Policlinico Universitario Duilio Casula, Azienda Ospedaliero-Universitaria di Cagliari, Cagliari, Medicina Interna, Allergologia ed Immunologia Clinica);*
- Giuseppe Montalto, Anna Licata, Filippo Alessandro Montalto, Angelo Rizzo, Silvia Como, Valentina Virzì *(Azienda Ospedaliera Universitaria Policlinico Paolo Giaccone, Palermo, UOC di Medicina Interna);*
- Giorgio Basile, Antonino Catalano, Federica Bellone, Concetto Principato, Angelo Cocuzza, Annamaria Buda, Fabio Malacarne, Francesco Corica *(Azienda Ospedaliera Universitaria Policlinico G. Martino, Messina, Unità Operativa di Geriatria);*
- Lorenzo Malatino, Benedetta Stancanelli, Valentina Terranova, Salvatore Di Marca, Rosario Di Quattro, Lara La Malfa, Rossella Caruso, Ivan Isaia, Federica Castelletti, Matteo Regolo, Clara Salmeri, Nicolas Cardaci, Alessandra Lanzafame *(Azienda Ospedaliera per l'Emergenza Cannizzaro, Catania, Clinica Medica Università di Catania);*
- Patrizia Mecocci, Carmelinda Ruggiero, Virginia Boccardi* (Università degli Studi di Perugia-Azienda Ospedaliera S.M. della Misericordia, Perugia, Struttura Complessa di Geriatria);*
- Pietro Minuz, Luigi Fondrieschi, Giandomenico Nigro Imperiale, Sarah Morellini, Andrea Sartorio *(Azienda Ospedaliera Universitaria Verona, Policlinico GB Rossi, Verona, Medicina Generale per lo Studio ed il Trattamento dell’Ipertensione Arteriosa);*
- Mario Pirisi, Gian Paolo Fra, Daniele Sola, Mattia Bellan *(Azienda Ospedaliera Universitaria Maggiore della Carità, Novara, Medicina Interna 1);*
- Emilio Simeone, Rosa Scurti, Fabio Tolloso *(Ospedale Civile Santo Spirito di Pescara, Geriatria);*
- Roberto Tarquini, Alice Valoriani, Silvia Dolenti, Giulia Vannini *(Ospedale San Giuseppe, Empoli, USL Toscana Centro, Medicina Interna I);*
- Sergio Harari, Chiara Lonati, Federico Napoli, Lisa Tescaro, Italia Aiello (*Clinica Medica a indirizzo cardio-respiratorio, Multimedica IRCSS, Milano*);
- Ferdinando Carlo Sasso, Teresa Salvatore, Lucio Monaco, Carmen Ricozzi, Francesca Coviello, Maria Elena Corona, Giovanni Di Lorenzo, Serafina Schettino, Christian Catalini, Davide Nilo (*Policlinico Università della Campania L. Vanvitelli, UOC Medicina Interna*);
- Alberto Pilotto, Ilaria Indiano, Silvia Podestà, Clarissa Musacchio. Lisa Cammalleri, Federica Gandolfo, Davide Gonella (*Ente Ospedaliero Ospedali Galliera Genova, SC Geriatria Dipartimento Cure Geriatriche, Neurologiche e Riabilitazione)*
- Moreno Tresoldi, Enrica Bozzolo, Sarah Damanti, Gaia Deonette, Giulia Marazzi, Rita Venezia, Lorenzo Ciocca, Jasmin Mahajne, Chiara Calabrese *(IRCCS Ospedale San Raffaele – Milano, Medicina Generale e delle Cure Avanzate);*
- Massimo Porta, Miriam Gino, Stefania Morra Di Cella, Bianca Pari, Edoardo Pace *(AOU Città della Salute e della Scienza di Torino – Torino, Medicina Interna 1U);*
- Patrizia Tilocca, Sebastiana Maria Atzori *(Azienda Ospedaliero Universitaria di Sassari– Sassari, Geriatria).*
- Alberto Moggi Pignone, Giulia Bandini, Anna Lo Cricchio, Maria Cristina De Santis, Paolo Mercatelli, Iacopo Bertoletti *(Azienda Ospedaliero Universitaria Careggi Firenze, Firenze, Medicina Interna 4);*
- Maria Lorenza Muiesan, Carolina De Ciuceis, Stefano Tenore, Fabio Cherubini, Francesco Russomanno *(Spedali Civili di Brescia, Brescia, Medicina Generale 2)*
- Nicola Liuzzi, Sokol *Rrodhe (Ospedale Civile “Edoardo Agnelli” Pinerolo - Torino, Medicina Generale);*
- Fiammetta Pagnozzi, Emanuela Messa, Francesca Gaia Bacchieri* (Ospedale Civico di Chivasso ASL Torino 4, Torino, S.C. Medicina Interna)*
- Gaetano Serviddio, Antonino Davide Romano* (Ospedali Riuniti di Foggia, Foggia, UOC Epatologia a Direzione Universitaria);*
- Gianluigi Vendemiale, Francesco Bellanti, Aurelio Lo Buglio* (Azienda Ospedaliero-Universitaria Policlinico Riuniti di Foggia, Foggia, UOC Medicina Interna e dell’Invecchiamento);*
- Antonino Tuttolomondo, Domenico Di Raimondo, Edoardo Pirera, Riccardo De Rosa *(Azienda Ospedaliera Universitaria Policlinico Giaccone Policlinico di Palermo, Palermo, UOC di Medicina Interna con Stroke Care);*
- Francesca Dassie, Angelo Di Vincenzo, Sara Brandolese*(Azienda Ospedaliera Università di Padova, Padova, UOC Clinica Medica III);*
- Carlo Custodero, Antonino Noto, Agostino Di Ciaula, Chiara Valentina Luglio, Anna Belfiore, Stefania Pugliese, Piero Portincasa *(Azienda Ospedaliero-Universitaria Policlinico di Bari, Bari, Medicina Interna Universitaria “Augusto Murri”);*
  Leonardo A. Sechi, Cristiana Catena, Gabriele Brosolo, Stefano Marcante, Andrea Da Porto, Luca Bulfone, Antonio Vacca *(Azienda Sanitaria Universitaria Friuli Centrale, Università di Udine, Dipartimento di Medicina, Clinica Medica, Udine).*

## Abbreviations

Re.Po.SI: Italian Society of Internal Medicine (SIMI) and the Mario Negri Institute of Pharmacological Research (Milan)
CIRS: Cumulative Illness Rating Scale (CIRS)
GDS-4: Geriatric Depression Scale (GDS-4)
BI: Barthel Index
SBT: Short Blessed Test
IQR: Interquartile ranges
vs: Versus
HR: Hazard ratio
ICD-9-CM: International Classification of Diseases, Ninth Revision, Clinical Modification
ATC: Anatomical Therapeutic Chemical Classification System
ADL: Activities of daily living
N: Number
P: Probability
IR: Incidence rate
CI: Confidence intervals
