# Supplementary material for: Association of the overlap of cognitive impairment and depression with 6-month mortality in hospitalized older adults: results from the Re.Po.SI register
Source: BMC Geriatr. 2025 Mar 18;25:185. doi: 10.1186/s12877-025-05818-8 (PMC11921589; doi:10.1186/s12877-025-05818-8)
Supplement: Supplementary file 1 — Supplementary Material 1. [file 12877_2025_5818_MOESM1_ESM.docx]

***Table S1***

***Demographics and characteristics of participants in the included sample compared to those excluded.***

|  | Total sample | Excluded | Final analytical sample | p |
| --- | --- | --- | --- | --- |
| Number of participants, n (%) | **8417** | 6461 | 1956 |  |
| Age, median [IQR] | **79.0 [73.0, 85.0]** | 79.0 [73.0, 85.0] | 80.0 [73.0, 85.0] | 0.026 |
| Sex = Male (%) | **4070 (48.4)** | 3103 (48.0) | 967 (49.4) | 0.291 |
| Education years, median [IQR] | **5.0 [5.0, 8.0]** | 5.0 [5.0, 8.0] | 5.0 [5.0, 8.0] | 0.071 |
| Civil status, n (%) |  |  |  | 0.424 |
| *married* | **2913 (53.7)** | 1912 (54.2) | 1001 (52.7) |  |
| *widowed* | **1971 (36.3)** | 1251 (35.5) | 720 (37.9) |  |
| *separated* | **93 (1.7)** | 64 (1.8) | 29 (1.5) |  |
| *divorced* | **93 (1.7)** | 61 (1.7) | 32 (1.7) |  |
| *single* | **353 (6.5)** | 237 (6.7) | 116 (6.1) |  |
| Living with = others (%) | **4059 (75.9)** | 2665 (76.4) | 1394 (75.1) | 0.308 |
| Alcohol consumption (%) |  |  |  | <0.001 |
| *Never* | **3920 (57.7)** | 2907 (59.5) | 1013 (53.1) |  |
| *ex-drinker* | **830 (12.2)** | 727 (14.9) | 103 (5.4) |  |
| *drinker* | **835 (12.3)** | 517 (10.6) | 318 (16.7) |  |
| *Social drinker* | **1210 (17.8)** | 738 (15.1) | 472 (24.8) |  |
| Collar^1^ = White (%) | **2261 (37.1)** | 1712 (36.8) | 549 (38.2) | 0.355 |
| P-ADL^2^ median [IQR] | **91.0 [66.0, 100.0]** | 90.0 [65.0, 100.0] | 91.0 [68.0, 100.0] | 0.889 |
| CIRS^3^ Illness Severity Index, median [IQR] | **1.6 [1.4, 1.8]** | 1.6 [1.4, 1.8] | 1.7 [1.5, 1.9] | <0.001 |
| CIRS^3^ Co-morbidity Index, median [IQR] | **3.0 [2.0, 4.0]** | 3.0 [1.0, 4.0] | 3.0 [2.0, 4.0] | <0.001 |
| Prescribed pharmaceuticals median [IQR] | **5.0 [4.0, 8.0]** | 5.0 [3.0, 7.0] | 6.0 [4.0, 8.0] | 0.028 |

^1.^ White-collar workers generally perform job duties in an office or other administrative settings.

^2.^ Personal Activities of Daily Living score with Barthel Scale.

^3.^ Cumulative Illness Rating Scale

MISSING

Sex, n (%) = 2(0.0)

Civil status, n (%) = 2994(35.6)

Living with, n (%) = 3070 (36.5)

Alcohol consumption, n (%) = 1622 (19.3)

Collar, n (%) = 2329 (27.7)

***Table S2***

***Hazard ratio (HR) for association between 6-month mortality and Cognitive Impairment, Depression, and their Interaction***

|  | **HR (95%CI) unadjusted** | **HR (95%CI) adjusted*** |
| --- | --- | --- |
| **Cognitive impairment** | 2.08 (1.63 – 2.67) | 1.94 (1.47-2.56) |
| **Depression** | 1.30 (1.00-1.71) | 1.37(1.03-1.84) |
| **Interaction between Cognitive impairment and depression** | 0.64(0.45-0.92) | 0.65(0.44-0.96) |

*Adjusted for: age, sex, years of education, prescribed pharmaceuticals and civil status

***Table S3***

***Sensitivity analysis at 3 months***

|  | Original Analysis n= 1956 | Alternative Analysis  n= 4780 | Original Analysis  n= 1956 | Alternative Analysis  n= 4780 |
| --- | --- | --- | --- | --- |
|  | IR | IR | Adjusted* HR | Adjusted * HR |
| No cognitive impairment, no depression | 43.3 | 33.7 | REF | REF |
| No cognitive impairment, depression | 55.9 | 45.7 | 1.33 | 1.32 |
| Cognitive impairment, no depression | 81.1 | 81.4 | 1.93 | 2.16 |
| Cognitive impairment, depression | 68.9 | 64.7 | 1.71 | 1.76 |

IR: incidence rate

HR: hazard ratio

*Adjusted for: age, sex, years of education, prescribed pharmaceuticals, civil status
